# An unusual giant schwannoma of cervical sympathetic chain: a case report

**DOI:** 10.1186/s13256-016-0812-z

**Published:** 2016-02-02

**Authors:** Taoufik Adouly, Choaib Adnane, Tarek Oubahmane, Sami Rouadi, Redallah Abada, Mohamed Roubal, Mohamed Mahtar

**Affiliations:** Department of ENT, Hopital 20 Août, Ibn Rochd University Hospital, Casablanca, Morocco

**Keywords:** Schwannoma, Cervical sympathetic chain, Clinical, Radiological, Treatment

## Abstract

**Background:**

Schwannomas are benign, well-differentiated tumors that originate from Schwann cells. Involvement of the cervical sympathetic nerve is relatively rare. Computed tomography is indispensable for the diagnosis. The treatment is surgical. Histological examination confirms the diagnosis. Horner’s syndrome postoperatively is supportive of the diagnosis. The rarity of giant cervical sympathetic chain schwannoma made the case of our patient interesting to report. Furthermore, our patient’s immense tumor size is very rare, and we could not find any similar report in the literature. Cervical sympathetic chain schwannoma is frequently confused with schwannoma of the vagus nerve on clinical and radiological examination, and its diagnosis can therefore be challenging for clinicians, radiologists, and pathologists.

**Case presentation:**

We report a rare case of cervical schwannoma in a 40-year-old Moroccan woman who presented with a large parapharyngeal mass. Computed tomography revealed a giant, heterogeneous, well-defined mass measuring 110 × 100 × 147 mm, occupying the right carotid triangle, and descending to the superior mediastinum. Surgical excision with a transcervical approach was done. Histological examination confirmed the diagnosis. The patient’s postoperative course was marked by Horner’s syndrome.

**Conclusions:**

Cervical sympathetic chain schwannoma is a rare, benign tumor. It should be considered in the differential diagnosis in patients presenting with a lateral neck mass. Surgical exploration must be discussed for a tumor with a large volume.

## Background

Schwannoma is a benign mesenchymal tumor exclusively developed from Schwann cells surrounding the peripheral nerve sheath. Schwannomas are found in 25–45 % of cases in the cervical area, mostly the vagus nerve. Schwannomas arising from the cervical sympathetic chain are very rare [1]. The treatment of choice is surgery, but forbearance should always be discussed with the patient. The patient should be informed of the risks of functional sequelae related to this surgery. Schwannomas with this presentation are frequently confused in clinical and radiological examination with schwannomas originating from the vagus nerve. Their diagnosis may be a challenge for clinicians, radiologists, and pathologists [2]. We discuss a recent case of a patient with a giant cervical sympathetic chain schwannoma, along with the radioclinical, histopathological, and therapeutic aspects of this rare tumor, as well as outcomes.

## Case presentation

We report a case of a 40-year-old Moroccan woman who consulted our hospital for immense swelling in the right neck area of 2 years’ duration and slowly increasing in size. She had no personal or family history of malignancy. She had slight dysphagia and dyspnea. She had no dysphonia, fever, syncopal attacks, compression complaints, or nasopharyngeal discomfort.

**Fig. 1:**
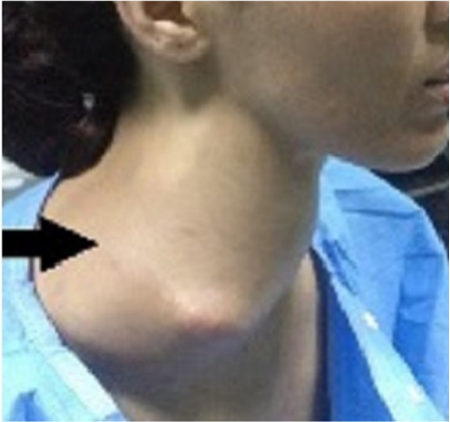
Objective right cervical swelling (*arrow*) measuring approximately 10 × 10 cm in the right carotid triangle

The patient’s physical examination revealed unusual swelling measuring approximately 10 × 10 cm in the right carotid triangle (Fig. 1). The swelling was firm, oblong, nonpulsatile, nontender, and not fixed to the overlying skin. The patient’s oropharyngeal examination showed a visible bulge in the right tonsil and posterior pillar embarrassing respiration and deglutition. Indirect laryngoscopy revealed that both vocal cords were mobile. The results of her nasal endoscopic and cranial nerve examinations were normal.

**Fig. 2:**
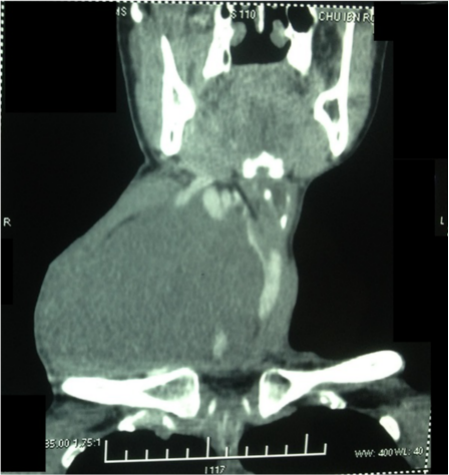
Coronal computed tomographic scan showing the patient’s cervical mass, which was huge, well-defined, measured 110 × 100 × 147 mm, and occupied the right carotid triangle

**Fig. 3:**
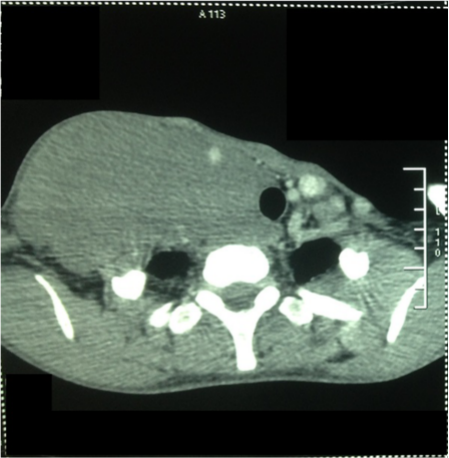
Axial computed tomographic scan showing the patient’s cervical mass, which was displacing the larynx, the trachea, and the thyroid gland outwardly

Computed tomography highlighted a huge, well-defined mass measuring 110 × 100 × 147 mm, occupying the right carotid triangle, and descending to the superior mediastinum. The mass was heterogeneous and pushing the carotid artery and internal jugular vein anteriorly. It was displacing the larynx, the trachea, and the thyroid gland outside. Contrast dye showed late contrast enhancement of the peripheral portion of the mass, with its center remaining isodense. No neck node was observed (Figs. 2 and 3). The results of fine-needle aspiration provided no additional information.

**Fig. 4:**
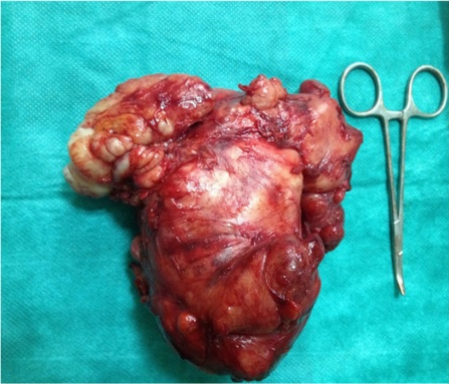
The patient’s excised tumor

A decision was made to perform surgical excision because the clinical results and computed tomography observations were deemed sufficiently suggestive of the diagnosis and in the absence of risk factors (alcohol, tobacco). Surgical excision was done using a transcervical approach with the patient under general anesthesia. A large tumor was found deep to the sternocleidomastoid muscle. It was separated from the surrounding structures by blunt dissection using a finger. The carotid artery and the internal jugular vein were displaced anteriorly but were not compressed. The mass appeared to have developed from the cervical sympathetic chain and not the vagus nerve. The mass was removed completely with difficulty after sacrificing some nerve fiber (Fig. 4).

**Fig. 5:**
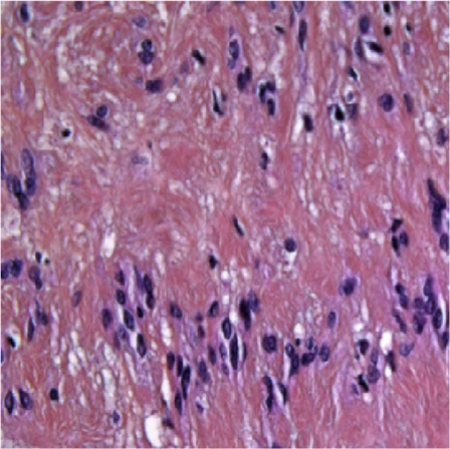
Histological slice showing cervical schwannoma (Antoni A appearance). The microscopic appearance of the tumor indicated it was composed of compact, broad, interlacing ribbons of extended spindle cells with elongated nuclei arranged in waves. Hematoxylin and eosin stain, original magnification ×400

After the operation, the patient was transferred to the intensive care unit. During the first day, a partial, well-tolerated Horner’s syndrome was observed, and the patient was treated with corticosteroids. No significant bleeding was seen. On the third day after surgery, the patient was found to have a rhythm disorder (arrhythmia). Her histopathological report showed a benign Antoni type A schwannoma originating from the cervical sympathetic chain (Fig. 5).

## Discussion

In 1899, Ritter reported the first observation of cervical schwannoma [3]. This pathological entity occurs in the head and neck in 25–45 % of patients [1, 3]. The commonest site is the parapharyngeal space, usually of vagal origin or in the last four cranial nerve roots and rarely from the sympathetic chain [2].

Cervical schwannomas usually occur between 20 and 70 years of age in both sexes [4]. The clinical presentation is not pathognomonic. The majority present as cervical swelling with asymptomatic, gradually increasing volume [4]. They sometimes may cause exerting pressure on the surrounding structures (for example, dysphagia) [5]. Features of nerve compression (preoperative Horner’s syndrome) are rare [6].

Radiological investigation is required for better management. Computed tomography allows determination of tumor size and extent to the parapharyngeal area, the degree of tumor vascularity, and the relationship of the tumor with the jugular vein and the carotid artery [6]. When the tumor originates from the vagus nerve, the mass increases the space between the internal carotid artery or common carotid artery and the internal jugular vein. A schwannoma that arises from the cervical sympathetic chain causes anterior displacement of vascular structures [4]. The mass is seen as hypodense with some degree of enhancement and well-delineated margins [6]. A schwannoma is usually hypointense on T1-weighted magnetic resonance images and hyperintense on T2-weighted magnetic resonance images [1]. In our patient, a huge mass measuring 110 × 100 × 147 mm was revealed. This immense tumor size is very rare, and we could not find any similar report in the literature.

The differential diagnosis includes lymph nodes, congenital cysts, paragangliomas of the vagus nerve, and carotid body tumors [7]. Fine-needle aspiration cytology is often noncontributory [3]. It allows the diagnosis of cervical schwannomas in just 25 % of cases [1].

Surgery is the treatment of choice for schwannomas. During surgery, we found that our patient’s lesion was positioned eccentrically to the nerve. Complete surgical removal of the mass, without sacrificing nerve fiber, is better and is possible only when the capsule is easily separable from the underlying fibers [7, 8]. In our patient, we could not remove the mass without sacrifice of some nerve fibers. A more common postoperative complication is Horner’s syndrome [6]. Our patient had partial well-tolerated Horner’s syndrome postoperatively.

Histological examination revealed that our patient’s tumor was encapsulated. Two microscopic patterns of schwannoma exist: Antoni A and Antoni B. Antoni type A is characterized by broad, interlacing ribbons of extended spindle cells with elongated nuclei arranged in waves, drifts, and whorls. Antoni type B is characterized by hypocellularity with a large quantity of myxoid tissue. In immunohistochemical analysis, S100 protein is positive in all schwannomas and neurofibromas [9]. In our patient, the mass was classified as type A according to Antoni’s classification.

## Conclusions

Our patient’s case illustrates that, although cervical sympathetic chain schwannoma is relatively rare, it should be considered in the differential diagnosis of benign and malignant lesions. In all cases, the patient should be advised of the occurrence of postoperative Horner’s syndrome, for which there is no effective treatment. The rarity of giant schwannoma makes our case interesting to report.

## Consent

Written informed consent was obtained from the patient for publication of this case report and any accompanying images. A copy of the written consent is available for review by the Editor-in-Chief of this journal.
